# Spinal Cord Injury Causes Reduction of *Galanin* and *Gastrin Releasing Peptid*e mRNA Expression in the Spinal Ejaculation Generator of Male Rats

**DOI:** 10.3389/fneur.2021.670536

**Published:** 2021-06-22

**Authors:** James W. Wiggins, Jonathan E. Sledd, Lique M. Coolen

**Affiliations:** ^1^Department of Neurobiology and Anatomical Sciences, University of Mississippi Medical Center, Jackson, MS, United States; ^2^Graduate Program in Neuroscience, University of Mississippi Medical Center, Jackson, MS, United States; ^3^Department of Biological Sciences, Kent State University, Kent, OH, United States

**Keywords:** contusion spinal injury, sexual dysfunction, anejaculation, lumbar spinal cord, urogenital

## Abstract

Spinal cord injury (SCI) in men is commonly associated with sexual dysfunction, including anejaculation, and chronic mid-thoracic contusion injury in male rats also impairs ejaculatory reflexes. Ejaculation is controlled by a spinal ejaculation generator consisting of a population of lumbar spinothalamic (LSt) neurons that control ejaculation through release of four neuropeptides including galanin and gastrin releasing peptide (GRP) onto lumbar and sacral autonomic and motor nuclei. It was recently demonstrated that spinal contusion injury in male rats caused reduction of GRP-immunoreactivity, but not galanin-immunoreactivity in LSt cells, indicative of reduced GRP peptide levels, but inconclusive results for galanin. The current study further tests the hypothesis that contusion injury causes a disruption of *GRP* and *galanin* mRNA in LSt cells. Male rats received mid-thoracic contusion injury and *galanin* and *GRP* mRNA were visualized 8 weeks later in the lumbar spinal cord using fluorescent *in situ* hybridization. Spinal cord injury significantly reduced *GRP* and *galanin* mRNA in LSt cells. *Galanin* expression was higher in LSt cells compared to *GRP*. However, expression of the two transcripts were positively correlated in LSt cells in both sham and SCI animals, suggesting that expression for the two neuropeptides may be co-regulated. Immunofluorescent visualization of galanin and GRP peptides demonstrated a significant reduction in GRP-immunoreactivity, but not galanin in LSt cells, confirming the previous observations. In conclusion, SCI reduced *GRP* and *galanin* expression in LSt cells with an apparent greater impact on GRP peptide levels. GRP and galanin are both essential for triggering ejaculation and thus such reduction may contribute to ejaculatory dysfunction following SCI in rats.

## Introduction

Spinal cord injury (SCI) is a devastating neural injury affecting a wide array of functions. Following initial stabilization after SCI, quality of life issues become increasingly important to patients. Surveys of patients with SCI have demonstrated that regaining sexual function is an important goal, even surpassing that for recovery of walking among paraplegic patients (1–3). This is especially notable considering over 90% of male patients suffering from SCI experience sexual dysfunction including anejaculation (1–16). Despite the need for better treatments for sexual dysfunction following SCI, advances are impeded by our lack of understanding of the effects of SCI on the spinal generators for sexual function (5–7, 13).

Ejaculation is a spinal reflex controlled by the spinal ejaculation generator located in the lumbosacral spinal cord (17, 18). The principle component of this generator is a population of neurons referred to as lumbar spinothalamic neurons (LSt) cells as cell-specific ablations of these cells completely disrupt ejaculatory behavior (17) and reflexes (19). Lumbar spinothalamic cells are located within spinal segments L3 and L4 surrounding the central canal in lamina X and the medial portion of lamina VII (17, 20). Even though LSt cells were named based on their projections to the parvocellular subparafascicular thalamic nucleus (21), they control ejaculatory reflexes via intraspinal connections, by integrating sensory inputs during mating to control the coordinated autonomic and motor output (17, 19). The exact role of the thalamic projections of LSt cells is unknown but hypothesized to contribute to reward associated with the ejaculatory reflex (22). Lumbar spinothalamic cells express the neuropeptides galanin, gastrin releasing peptide (GRP), enkephalin and cholecystokinin (CCK) (17, 22–27), and trigger ejaculation via the release of these neuropeptide onto preganglionic and motor nuclei (26–28). Anatomically, LSt cells have axonal projections co-containing these neuropeptides to sympathetic and parasympathetic preganglionic cells in intermediolateral cell column, dorsal central nucleus, and sacral parasympathetic nucleus, respectively, and pudendal motor neurons in sacral nucleus of bulbocavernosus (17, 24). It has been shown that LSt cells also comprise the human spinal ejaculation generator, which is thus anatomically identical in human and rat (18). The clinical outcome of ejaculatory function in SCI patients has been directly correlated with the degree of intactness of the region containing the spinal ejaculation generator (especially L3–5) of the spinal cord (11), further confirming cross-species homology of the spinal ejaculation generator location. Importantly, LSt cells express galanin in humans as in rats (18), suggesting that the LSt neuropeptides may have a similar role in controlling ejaculation in human as in rat. Therefore, we have proposed the central hypothesis that SCI disrupts the synthesis of neuropeptides within LSt cells, resulting in loss of neuropeptide release and in turn causing impaired ejaculatory reflexes.

In order to test this hypothesis in male rats, we have previously shown that SCI caused ejaculatory dysfunction in male rats, similar to humans (29). Specifically, ejaculatory reflexes were examined in anesthetized male rats with chronic contusion spinal injury at mid thoracic spinal levels. The contusion injury model uses a single brief displacement of the spinal cord and has been demonstrated to closely replicate the key pathophysiological features of human injury by producing either prolonged, or rapidly applied, extradural compression (30–32). Ejaculatory reflexes in chronic mid-thoracic contusion-injured male rats were determined after stimulation of the dorsal penile nerve, which relays the sensory inputs needed to trigger ejaculation in rats (19) and in other mammals including apes and humans (33–35). Indeed, it was shown that sensory stimulation was unable to trigger ejaculatory reflexes in SCI males (29). This was a critical observation as previous studies had shown effects of select spinal lesions or lateral hemisections on erectile and pudendal nerve reflexes (36–38), but detailed analyses of ejaculatory reflexes following chronic SCI and following a contusion injury had not been previously described in rodents. Moreover, in our previous study, ejaculatory reflexes were examined after removal of all remaining supraspinal inputs to the spinal ejaculation generator, hence anejaculation was independent of changes in supraspinal input and likely caused by long-term alterations within the spinal ejaculation generator (29).

To test the hypothesis that SCI impact synthesis and release of LSt peptides, we previously evaluated changes in galanin and GRP immunoreactivity in LSt cells (39). It was demonstrated that chronic mid-thoracic contusion injury selectively reduced immunoreactivity for GRP in LSt cells, whereas there was a failure to detect reduction of immunoreactivity for galanin. These findings are indicative of reduced GRP peptide levels in LSt cells after SCI. The finding that galanin was unaffected was surprising and may suggest that SCI caused modifications specifically of the *GRP* gene without affecting the *galanin* gene. However, another probable hypothesis is that galanin peptide levels may indeed have been affected by SCI, but the previous study failed to detect such changes due to potentially high levels of galanin neuropeptide in LSt cells and the limited sensitivity of immunofluorescence visualization as a quantitative assay for peptide detection. Therefore, to address these two hypotheses, the current study utilized Fluorescent *In Situ Hybridization* to analyze the impact of chronic SCI on mRNA expression levels for *galanin* and *GRP* in LSt cells of male rats with chronic contusion injury at mid-thoracic spinal cord.

## Methods

### Animals

Adult male Sprague Dawley rats (*n* = 10; Charles River, Wilmington, MA, 225–250 g) were pair housed in in standard Plexiglas cages. Animals were housed in humidity and temperature-controlled rooms on a 12-h light/dark cycle with lights off at 10 a.m. Food and water was freely available. All studies were carried out in accordance with the National Institutes of Health guidelines involving vertebrate animals in research and were approved by the Institutional Animal Care and Use Committee at the University of Mississippi Medical Center.

### Contusion Injury Surgeries

Rats were randomly divided into SCI (*n* = 5) and sham (*n* = 5) groups. All rats (sham and SCI) were deeply anesthetized with ketamine/xylazine (83 and 13 mg/kg, respectively, 1 ml/kg i.p. injection) and the backs were shaved and cleaned with betadine and ethyl alcohol (70%). A midline incision was made over thoracic and lumbar segments, muscle and connective tissue surrounding the spinal column at T6–7 segments were carefully dissected, and laminectomy of T6 and T7 vertebra was performed. Rats were placed on the base platform of the IH-400 impactor (Precision Systems and Instrumentation, LLC, Lexington, KY) and their spinal columns were stabilized using forceps attached to the platform. Spinal cord injury, but not sham, animals received a contusion injury at the T6–T7 spinal levels of the spinal cord, using the IH-400 impactor with the tip of the impact rod positioned 5 mm above the exposed cord. Software parameters were selected to deliver a force of 200 Kdyn applied to the dura of the exposed spinal cord with a dwell time of 0 s. Actual force delivered ranged from 201 to 216 Kdyn (Mean: 209 ± 2.83 Kdyn). Spinal cord displacement values ranged from 1,111 to 1,270 μm (Mean: 1,178 ± 28.7 μm). Velocity of the impact rod ranged from 117 to 120 mm/s (Mean: 119 ± 0.8 mm/s). Sham animals received all treatments except the contusion injury. After surgery, the layers of muscle and skin were sutured separately, with absorbable sutures (Ethicon, Mokena, IL, Cat# J415) and wound clips (9 mm; World Precision Instruments, Sarasota, FL, Cat# 500345), respectively. All animals (SCI and sham) received subcutaneous injections (s.c.) of the analgesic carprofen (5 mg/ml/kg), and intamuscular (i.m) injections of the antibiotic Baytril (5 mg/ml/kg; Bayer Pharmaceuticals, Leverkusen, Germany, Cat# RXBAYTRIL) immediately prior to surgery and for 3 days post-operation. Bladder dysfunction was present in all SCI animals and bladders were emptied by light manual compression twice per day until bladder function returned. Sham animals were handled without bladder expression.

### Locomotor Testing

Locomotor assessment was performed as described in our previous publications (29, 39) at 3, 4, and 7 weeks after surgery to confirm effects of SCI. Briefly, locomotor activity was measured during the dark phase using an Open Field Apparatus (Med Associates Inc., St. Albans City, VT) surrounded by two 16 × 16 photobeam arrays for detection of vertical and horizontal movement. Locomotor activity was measured during a 15-min period and presented as ambulatory distance in the horizontal plane (horizontal distance in cm) and time spent rearing (vertical time in s). Statistical comparisons were conducted using student *t*-tests for each weekly test and Sham and SCI animals differed statistically in both ambulatory distance and vertical time (Table 1). In addition, activity analysis using the Basso, Beattie, and Bresnahan scale (BBB) (40) was conducted at 4 weeks following surgery and scores different significantly (*p* < 0.001) between Sham (Score 21 ± 0) and SCI (Score 10.6 ± 1.2).

**Table 1:** Locomotor activity in Sham and SCI male rats shown as locomotor movement in the horizontal plane (horizontal distance in cm) and time spent rearing [vertical time in seconds (s)].

		**Horizontal distance (cm)**	**Vertical time (s)**
Week 3	Sham	3,170 ± 750	271 ± 43
	SCI	1,181 ± 161^*^	205 ± 59
Week 4	Sham	2,357 ± 416	179 ± 31
	SCI	1,351 ± 229^*^	94 ± 19^*^
Week 7	Sham	1,841 ± 247	156 ± 16
	SCI	969 ± 135^*^	88 ± 18^*^

* *Indicates significant differences between sham and SCI in ambulatory distance (Week 3: p = 0.0159, Week 4: p = 0.033; Week 7: p = 0.0135) and vertical time (Week 4: p = 0.0248; Week 7: p = 0.0109)*.

### Perfusion and Tissue Processing

Eight weeks after contusion or sham surgery, animals were overdosed with sodium pentobarbital (390 mg/ml/kg bodyweight, Fatal Plus, Patterson) and were perfused transcardially first with 10 ml 0.9% sodium chloride (saline; Fisher Cat#BP-358-212) and then 500 ml of 4% paraformaldehyde in in 0.1 M sodium phosphate buffer (Electron Microscopy Sciences, Hatfield, PA, Cat# 19210). Spinal cords were removed and post-fixed in the same fixative for 24 h, then placed in gradated series of sucrose solutions [10, 20–30% in RNAse free phosphate-buffered saline (0.1 M PB with 0.9% sodium chloride; PBS) with 0.01% sodium azide, Sigma]. Each lumbosacral spinal cord was blocked into small segments, preserving the rostral-caudal orientation, placed in a row onto the cryostat sample holder, and sectioned at 14 μm thickness using a Cryostat (Leica model 3050S, Nussloch Germany). Rows of sections containing different levels of lumbosacral spinal cord were collected on glass microscope slides (Superfrost; Fisher Scientific, Cat: 12-550-15, Pittsburgh, PA). Each slide contained three serial rows of section that together spanned 42 μm. Sections were stored at −80°C until further processing.

### *In situ* Hybridization

Fluorescent *in situ* hybridization was conducted using RNAscope® Fluorescent Multiplex Reagent Kit V2 (Advanced Cell Diagnostics, Catalog No. 323100, Lot No: 2003230. Newark, CA). RNAscope target probes specific for rat *galanin* mRNA (Advanced Cell Diagnostics, REF No: 440681, Lot No: 18085A) and rat *gastrin releasing peptide* mRNA (Advanced Cell Diagnostics, REF No: 497781-C3, Lot No: 18085A) were used. Positive (Advanced Cell Diagnostics, REF No: 320891, Lot No: 17065A) and negative (Advanced Cell Diagnostics, REF No: 320871, Lot No: 17026A) control probes were included in each experiment. All *in situ* and amplification steps took place in a HybEZ™ humidified oven (Advanced Cell Diagnostics, Catalog No. 241000ACD, Newark, CA) and slides were washed in RNAscope® 50X Wash Buffer (Advanced Cell Diagnostics, REF No: 320058, Lot No: 2002627, 2 min at room temperature) between all steps. Instructions by Advanced Cell Diagnostics for Fluorescent Multiplex Reagent Kit V2 were followed with minor modifications. First, slides with sections were heated at 60°C (1 h), submerged in chilled 4% paraformaldehyde (at 4°C, 1 h), incubated in Hydrogen Peroxide solution (Advanced Cell Diagnostics, REF No: 322335, Newark, CA, 10 min at room temperature), treated with Target Retrieval solution (Advanced Cell Diagnostics, REF No: 322001, 15 min at 98°C), washed in 100% ethanol, and air dried. A hydrophobic barrier was created around the tissue using an Immedge™ hydrophobic barrier pen (Vector, Cat No. H-4000, Burlingame, CA). Sections were treated with RNAscope® Protease III (Advanced Cell Diagnostics, REF No: 322337, 30 min at 40°C), and subsequently incubated with RNAscope target and control probes (2 h), and subsequently with 4–6 drops of RNAscope® Multiplex FL v2 Amp 1 (Advanced Cell Diagnostics, REF No: 323101, Newark, CA, 30 min), followed by FL v2 Amp 2 (Advanced Cell Diagnostics, REF No: 323102, 30 min), then FL v2 Amp 3 (Advanced Cell Diagnostics, REF No: 323103, 30 min), followed by FL v2 HRP-C1 (Advanced Cell Diagnostics, REF No: 323104, 15 min). Sections were next incubated with 120 μl per slide of TSA + Cy3 [Perkin Elmer, Catalog No: FP1170012UF, Waltham, Massachusetts, 1:1,500 concentration in RNAscope® TSA buffer (Advanced Cell Diagnostics, REF No: 322809, Newark, CA, 30 minutes)], RNAscope® Multiplex FL v2 HRP Blocker (4–6 drops, Advanced Cell Diagnostics, REF No: 323107, 15 min), and RNAscope® Multiplex FL v2 HRP-C3 (Advanced Cell Diagnostics, REF No: 323106, 15 min). Sections were then incubated with 120 uL TSA + Fluorescein (Perkin Elmer, Catalog No: FP1168015UG, 1:1,500 in RNAscope® TSA buffer, 30 min), followed by 4–6 drops of RNAscope® Multiplex FL v2 HRP Blocker (Advanced Cell Diagnostics, REF No: 323107, 15 min). Finally, tissue was counterstained with DAPI (Advanced Cell Diagnostics, REF No: 323108, 30 s at room temperature), and coverslipped with ProLong Gold Antifade Mountant (Invitrogen REF No: P36930/P36934, Eugene, OR) and stored at 4°C.

Due to limited capacity of 24 slides in the HybEZ™ humidified oven, slides were divided over two separate runs. Each run included one or more slides of all animals in the study and were identical in all protocol steps. This resulted in three slides with at least one L3 and L4 section per slide for each animal. Each run also included one sham and SCI slide treated with the positive and negative control probes.

### RNAscope Analysis

#### Confocal Microscopy

**Figure 1:**
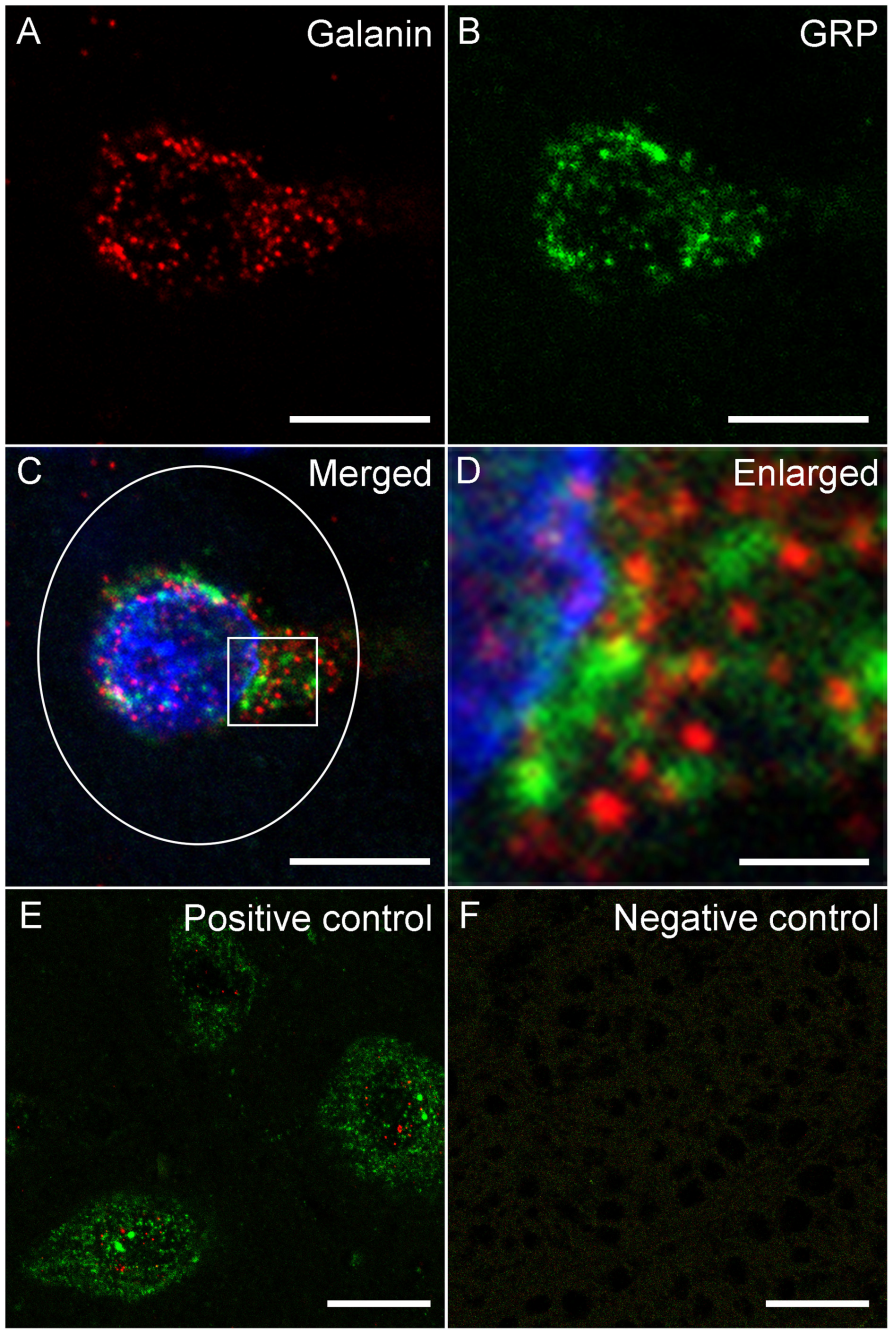
Fluorescent *in situ* hybridization for *galanin* and *GRP* mRNA. Representative confocal images (1 μm optical section) showing fluorescent signal for *galanin* **(A)** mRNA and *GRP* **(B)** mRNA in a single LSt cell, co-labeled with DAPI **(C)**. White ellipse shown in C is representative of the region of interest used for optical density analysis. Boxed area in **(C)** is shown enlarged in **(D)** demonstrating that *galanin* and *GRP* mRNA particles are distinct. **(E)** Positive control probe showing labeling for *POLR2A* (Red) and *UBC* (Green) mRNA. **(F)** Negative control probe showing lack of labeling. Scale bars represent 10 μm **(A–C)**, 2 μm **(D)**, and 25 μm **(E, F)**.

Slides were analyzed within 2–3 weeks after RNAscope. All cells in L3 and L4 spinal levels in laminae X and medial VII (i.e., LSt cells) of each animal that were expressing fluorescence for *galanin* (Figure 1A) and/or *GRP* (Figure 1B) mRNA were imaged using a Nikon D-Eclipse C1 laser-scanning confocal system (Nikon Corp, Minota, Tokyo, Japan) attached to a Nikon Eclipse E800 microscope (Nikon Corp). DAPI label was also acquired in a third channel (Figures 1C,D). Fluorophores were detected by three lasers at wavelengths of 408, 488, and 543 nm. Wavelengths were filtered by two dichroic mirrors filtering at 561 and 638 nm. Confocal z-stacks of optical images (1 μm optical sections taken at 60X magnification at 2X zoom) were obtained through labeled cell soma using EZ-C1 Gold version 3.80 software (Nikon Corp). Acquisition settings were identical for all images, for all cells in all animals. In addition, confocal images were obtained of cells in the superficial layers of the dorsal horn at L3 and 4 levels with fluorescent signal for *galanin* or *GRP* mRNA. Again, identical acquisition settings were used for all dorsal horn images, but the settings were optimized for the lower signal in dorsal horn cells and different from image settings for LSt cells. Positive and negative probe-control slides were examined for appropriate expression, but not included in quantitative analyses. In all runs, positive control slides demonstrated the expected fluorescent signal indicating the integrity of mRNA in the samples (Figure 1E), while negative control probes showed a lack of any signal demonstrating the specificity of the protocol (Figure 1F).

#### Optical Density Analysis

Confocal images containing LSt or dorsal horn cells were opened in Image J (ImageJ 1.51 w) and converted to 8-bit images. First, a threshold intensity for the optical density analysis was determined: an auto-threshold was recorded for each of the two channels corresponding to *galanin* and *GRP* on all optical slices in all images for all animals, for LSt cells and dorsal horn separately. An average of these thresholds was then determined for each of the two channels and used as the fixed threshold intensity for the optical density analysis. To determine the number of pixels above threshold, three optical slices were selected through the middle of each neuron expressing any level of *galanin* or *GRP*. The middle of the cell was determined by visualization of the nucleus in the DAPI-channel (Figure 1C). The number of pixels above threshold was measured using an ellipse with a width of 220 pixels and a height of 260 pixels (Total area 44,940 pixels) placed directly over the soma and recorded for each cell (Figure 1C). In addition, measurements of background regions were also recorded in the same images by placing the ellipse adjacent to the analyzed neuron and this background number of pixels above threshold was subtracted from the number of pixels above threshold in the labeled soma to provide the final value of pixels above background. Values were averaged over the three optical slices per cell and values for cells were averaged per animal for statistical comparisons between groups. This provided a range of 12-48 *galani*n-expressing LSt cells per animal for optical density analysis (mean numbers of cells per animal included in analysis: Sham L3: 11.4 ± 2.6, L4: 18.4 ± 3.5; SCI L3: 9.6 ± 2, L4: 14.6 ± 1.9 cells).

### Immunofluorescence

Two slides with sections for each animal were treated with 1% H_2_0_2_ (Fisher, Cat# H323-500) for 10 min and antibody incubation solution [saline-buffered sodium phosphate buffer (PBS)] containing 0.1% bovine serum albumin (Fischer, Cat# BP1605-100) and 0.4% Triton X-100 (Fisher, BP151-100). Next, sections were incubated with a polyclonal rabbit antibody specifically recognizing GRP (Phoenix, RRID: AB_2722600, Cat: H-027-12, Burlingame, CA; 1:20,000 in incubation solution; 17 h), biotinylated Donkey anti-rabbit (Jackson, Cat: 711065152, West Grove, PA; 1:500 in incubation solution; 1 h), ABC-elite (Vector, Ref: PK-6100, Burlington, CA; 1:1,000 in PBS; 1 h), tyramine sample amplification (Perkin Elmer, Ref: SAT700B001EA; Walthalm, MA; 1:250 in PBS + 1 μl/ml of 3% H_2_0_2_; 10 min), and with Dylight 488-conjugated streptavidin (Invitrogen; Cat: SA5-10086; Rockford, IL; 1:200; 30 min). Subsequently, sections were incubated with a polyclonal rabbit antibody specifically recognizing galanin (Peninsula, RRID: AB_518348, Cat: T4334, San Carlos, CA; 1:3,000; 17 hours) and Dylight 550-conjugated donkey anti-rabbit (Invitrogen; Cat: SA5-10039; Rockford, IL; 1:100, 30 min).

### Antibody Controls

Validation of the antibody against GRP was accomplished with pre-absorption with GRP peptide (Phoenix, #027-13; 10 μg/ml) which prevented all immunostaining. The galanin antibody has been validated previously (19–21, 26, 27, 41, 42).

### Immunofluorescence-Labeled Cell Analysis

The numbers of cells immunoreactive for galanin, GRP, or both were recorded using fluorescent microscope (Leica, CTR 500) by an observer blinded to experimental treatments. For each animal, all sections were examined and numbers of immunoreactive cells were recorded. Total numbers of cells were calculated for each animal. There were no differences in numbers of sections containing galanin, GRP, or dual-labeled cells between groups (Mean number of lumbar sections per animal included in analysis: Sham: 8.8 ± 1.2 SCI: 8.6 ± 0.7) but cells were expressed as cells/section in order to eliminate any effects of differences in numbers of analyzed sections between animals.

### Statistical Analyses

Optical density measurements of fluorescence signal for either galanin or GRP were compared between treatment groups using *t*-tests for L3 and 4 levels combined, or by two-way ANOVA with factors spinal level (L3 and L4) and treatment (SCI and sham) followed by Fisher LSD with 95% confidence levels. To evaluate if levels of galanin and GRP mRNA were correlated within individual cells a Pearson product-movement correlation was performed (SigmaPlot 14, Systat Software, San Jose, CA) using a confidence interval of 95%. Comparisons between groups for cell counts of immunofluorescent sections were conducted using Fisher LSD tests with 95% confidence levels.

## Results

### SCI Reduced Expression of *Galanin* and *GRP* mRNA in LSt Cells

**Figure 2:**
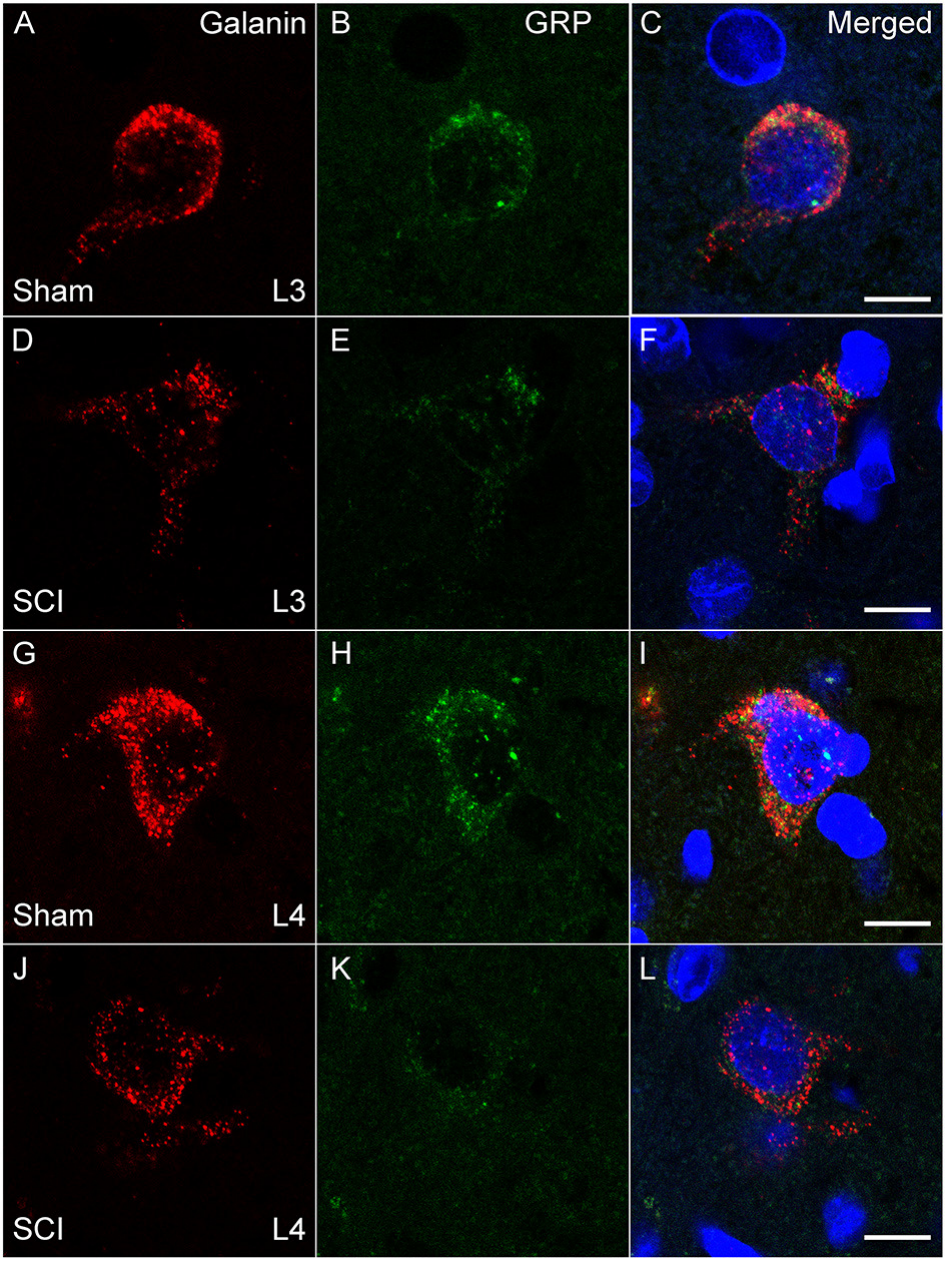
*Galanin* and *GRP* mRNA in LSt cells is reduced in SCI males. Representative confocal images (1 μm optical section) in a Sham **(A–C, G–I)** and SCI **(D–F, J–L)** animal showing SCI-induced reduction of fluorescent signal for *galanin* (**A, D, G, J**; Red) and *GRP* (**B, E, H, K**; Green) mRNA, in LSt cells in both L3 **(A–F)** and L4 **(G–L)** spinal levels. Merged images with DAPI are shown in **(C, F, I, L)**. Scale bars represent 10 μm.

**Figure 3:**
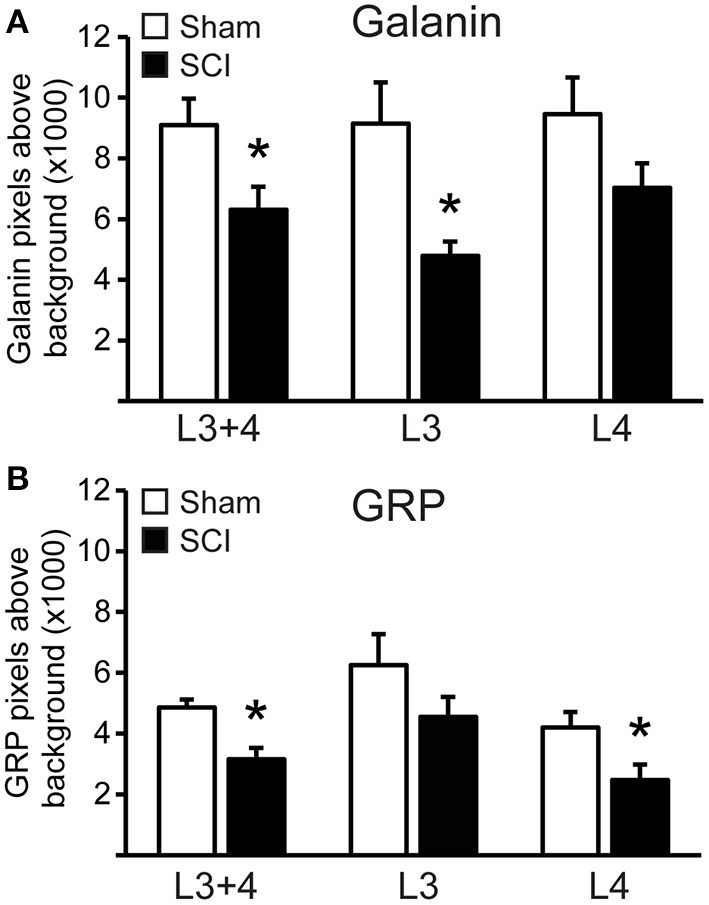
SCI reduced *galanin* and *GRP* mRNA in LSt cells. Quantitative analysis of fluorescent signal for *Galanin* **(A)** and *GRP* **(B)** mRNA in LSt cells in Sham (white bars) and SCI (black bars) groups. Data are expressed as mean ± SEM pixels above background for LSt cells in combined L3 and L4 spinal levels, and separately for L3 or L4 spinal levels. *Indicates statistically significant difference from Sham.

The distribution of cells co-localizing fluorescent signal for *galanin* and *GRP* mRNA paralleled that observed using immunohistochemistry (17, 20, 24, 26) and immunofluorescent labeling (39) in our previous publications. All *galanin* and *GRP* co-expressing cells were located lateral to the central canal at L3 and L4 lumbar levels in laminae X and medial laminae VII, as described previously using immunofluorescence (26, 39). There was no significant effect of SCI on the numbers of *galanin* and *GRP* co-expressing cells that were detected in the series of sections included in the analysis (mean number of cells per animal in L3+4 spinal levels: Sham: 29.8±5.4 and SCI: 24.2 ± 3.6). However, quantitative analysis of the fluorescence signal for *galanin* and *GRP* mRNA within these cells demonstrated that SCI caused significant reductions of both *galanin* (*p* = 0.043) and *GRP* (*p* = 0.006) mRNA expression in the population of LSt cells (L3+4 spinal levels; Figures 2, 3). In order to determine if SCI reduced transcript level similarly in L3 and L4 spinal levels, additional analysis was conducted for L3 and L4 LSt cells separately (Figure 3). Two-way ANOVA [Factors SCI (SCI-Sham) and spinal level (L3–L4)] revealed a main effect of SCI on *galanin* and *GRP* fluorescent signal [*Galanin*: *F*_(1, 16)_ = 11, *p* = 0.004; *GRP*: *F*_(1,16)_ = 5.92, *p* = 0.027] and a main effect of spinal level on *GRP* fluorescent signal [*F*_(1,16)_ = 8.55, *p* = 0.01], but not galanin. No interactions between SCI and spinal level were observed. Pairwise comparison revealed significant reductions in *galanin* fluorescent signal in L3 spinal segments (*p* = 0.016) and in *GRP* in L4 spinal level (*p* = 0.042) in SCI animals compared to sham. In SCI males, but not sham, *GRP* optical density was lower in L4 compared to L3 spinal segment (*p* = 0.038). Another observation was that fluorescent signal for *galanin* mRNA appeared stronger than that for *GRP* mRNA (Figure 3). Indeed, pixels above background were on average 4.9- (Sham) and 4.8- (SCI) fold higher for *galanin* than *GRP* (*p* = 0.0018 Sham; *p* = 0.0056 SCI), with no effect of SCI.

### *Galanin* and *GRP* mRNA Expression Correlates Within Cells

**Figure 4:**
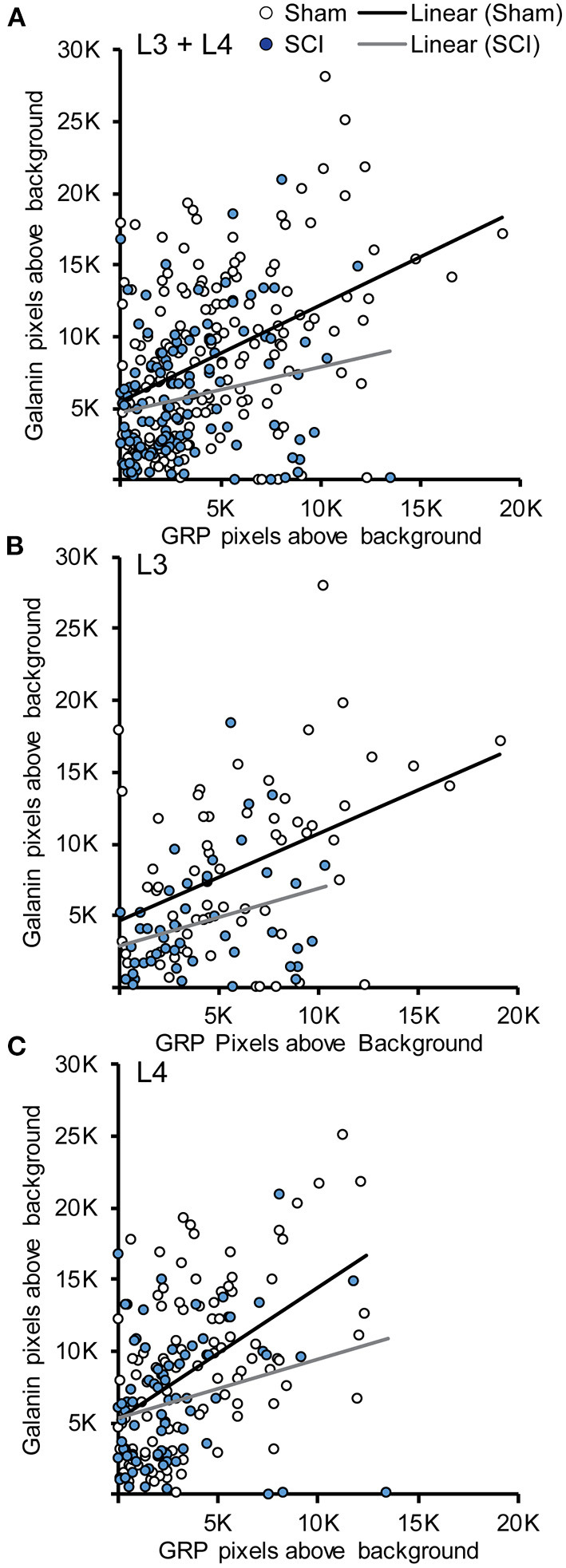
Association between *Galanin* and *GRP* mRNA in LSt cells. Scatterplots demonstrating the positive associations between fluorescent signal for *galanin* and *GRP* mRNA within LSt cells in combined L3 and L4 spinal levels **(A)**, and separately for L3 **(B)** or L4 **(C)** spinal levels. Each circle represents the fluorescent pixels above background for *galanin* and *GRP* within one cell. Cells of sham animals are shown in white open circles, cells of SCI animals in filled blue circles. Lines show the linear relationship based on Pearson Product-Movement Correlation analyses for Sham (black line) and SCI (gray line) groups.

An advantage of using dual fluorescent *in situ* hybridization is that it allows for a direct analysis of the association *galanin* and *GRP* transcripts within LSt cells and the effects of SCI on such an association. A Pearson product-moment correlation coefficient showed that fluorescent signal for *galanin* and *GRP* mRNA were positively correlated within cells of sham animals in both L3 and L4 spinal levels, indicating that in general cells with higher levels of *galanin* also expressed higher levels of *GRP* (L3+L4: *r* = 0.424, *p* < 0.001, *n* = 167 cells; L3: *r* = 0.429, *p* < 0.001, *n* = 64 cells; L4: *r* = 0.491, *p* < 0.001, *n* = 103 cells; Figure 4). Spinal cord injury reduced overall levels of both *galanin* and *GRP* mRNA, but the positive correlation between transcripts remained intact (L3+L4: *r* = 0.213, *p* = 0.017, *n* = 125 cells; L3: *r* = 0.312, *p* = 0.031, *n* = 48 cells; L4: *r* = 0.252, *p* = 0.027, *n* = 77 cells; Figure 4). Finally, analysis of both transcripts within LSt cells again showed that signal for *galanin* was greater compared to *GRP* (*p* < 0.001 for Sham and SCI).

### SCI Reduced *Galanin* mRNA Expression in Dorsal Horn

**Figure 5:**
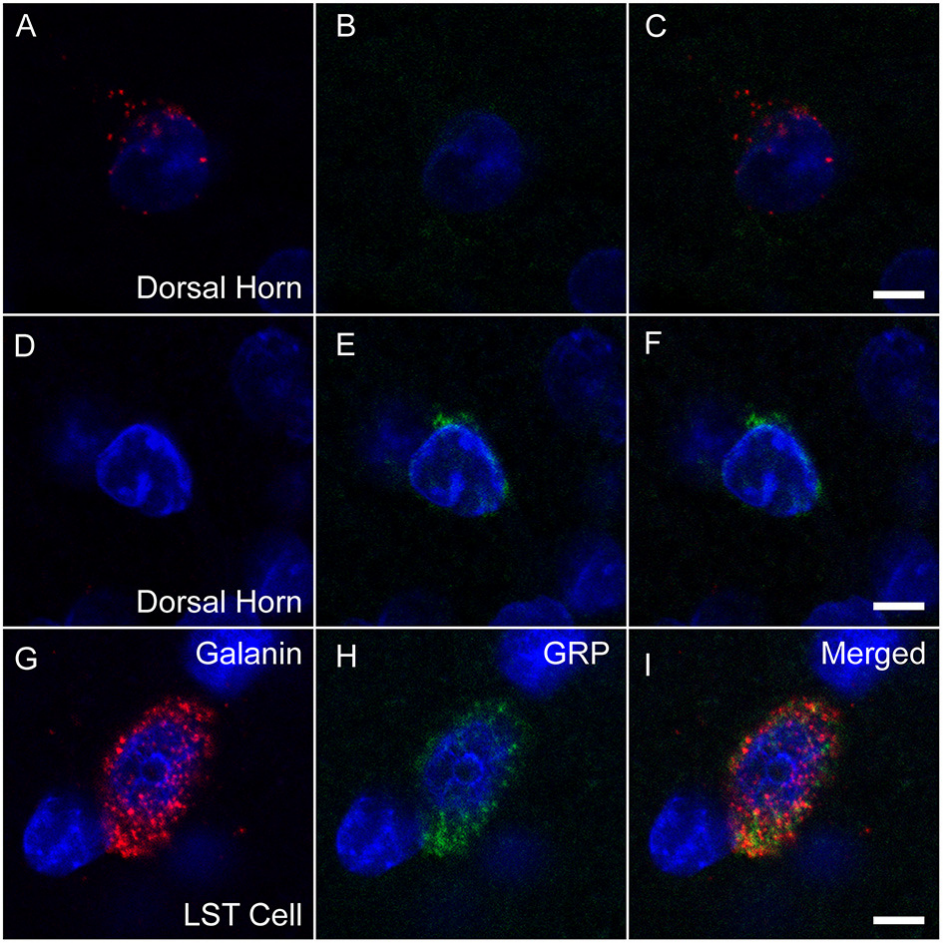
*Galanin* and *GRP* mRNA in LSt cells and dorsal horn neurons. Representative confocal images (1 μm optical section) showing fluorescent signal for *galanin* mRNA (red; **A, D, G**) or *GRP* (green; **B, E, H**) mRNA co-labeled with DAPI in blue in dorsal horn cells (**A–C, D–F**), while *galanin* and *GRP* are co-expressed in a LSt cell **(G–I)**. Merged channels are shown in **(C, F, I)**. Scale bars represent 5 μm.

*galanin* mRNA-expressing neurons were present in the dorsal horn of L3 and 4 levels (Figures 5D–F), but none of these neurons co-expressed *GRP*. Moreover, numbers of cells were low (mean number per animal: Sham: 5 ± 1.4; SCI: 3.8 ± 0.73) and fluorescent signal for *galani*n appeared much lower compared to that in LSt cells (Figures 5A–C). However, quantitative analysis showed that SCI caused a significant reduction in *galanin* mRNA fluorescence signal in L3+4 dorsal horn cells (Sham: 1,844 ± 61.9; SCI: 1,262 ± 218.1 pixels above background; *p* = 0.03). A few *GR*P mRNA expressing cells were also noted in dorsal horn but only at upper lumbar levels (L1-2) and in low numbers and were therefore not included in analysis (Figures 5G–I). None of these cells co-expressed *galanin* and fluorescent signal for *GRP* also appeared much lower compared to LSt cells (Figures 5A–C).

### SCI Reduced Immunoreactivity for GRP in LSt Cells

**Figure 6:**
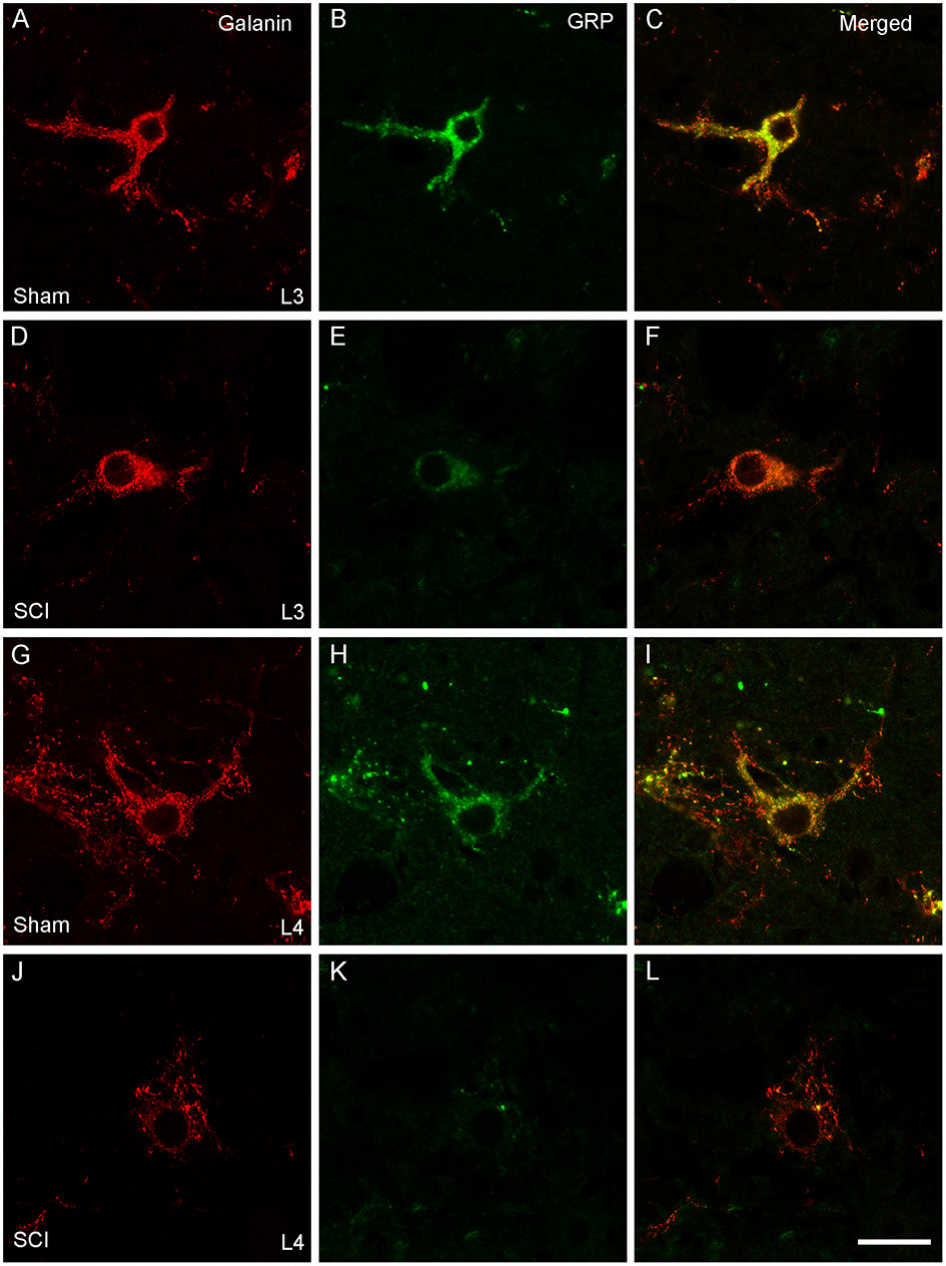
Galanin and GRP immunofluorescence in LSt cells. Representative confocal images (1 μm optical section) showing immunofluorescent labeling of galanin (red; **A, D, G, J**) and GRP (green; **B, E, H, K**) in LSt cells of sham **(A–C**, **G–I)** and SCI **(D–F**, **J–L)** males in L3 **(A–F)** and L4 **(G–L)** spinal levels. Merged channels are shown in **(C, F, I, L)**. Scale bar represent 25 μm.

**Figure 7:**
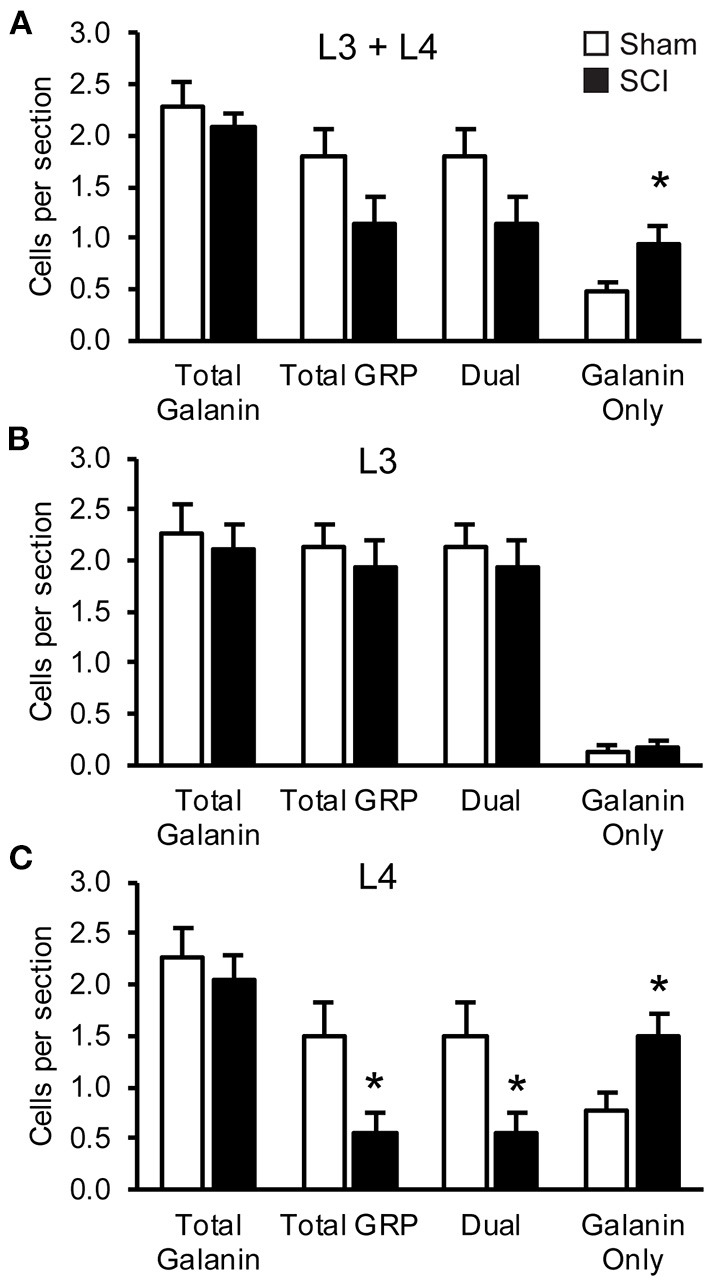
SCI reduced GRP-immunofluorescence in LSt cells. Quantitative analysis of numbers of cells immunofluorescently labeled for galanin or GRP. Analyses were based on cells in combined L3 and L4 spinal levels **(A)**, or separately for either L3 **(B)** or L4 **(C)** spinal levels in Sham (white bars) and SCI (black bars) groups. Data are expressed as mean ± SEM numbers of cells per section for the total numbers of galanin-ir cells (Total galanin; independent of GRP-ir) and GRP-ir cells (Total GRP; independent of galanin-ir). Analysis of the co-localization of galanin and GRP-immunoreactivity within LSt cells is shown as numbers of dual-labeled (Dual), or galanin-only labeled cells (Galanin only). GRP-only labeled cells weren't detected and hence not included on the graphs. *Indicates statistically significant difference from Sham. Data show that SCI did not reduce total numbers of galanin-ir LSt cells but reduced GRP-ir in LSt cells, resulting in fewer dual-labeled cells and more galanin-only LSt cells. This effect was most notable in L4 spinal levels.

In our previous study (39) we demonstrated that SCI severely reduced or eliminated GRP immunoreactivity (-ir) in LSt cells, while galanin was apparently not impacted. Specifically, the previous study showed that the numbers of galanin-ir LSt cells were unaltered by SCI, but significantly fewer galanin-ir LSt cells co-localized GRP-ir compared to sham controls. The current finding that SCI reduced mRNA expression for both *galani*n and *GRP* transcripts is in apparent contrast to those earlier findings. In addition, the current study investigated mRNA expression at 8 weeks after SCI, while the previous immunofluorescence study determined changes at 5–6 weeks after SCI. Therefore, analysis of immunofluorescence labeling for galanin and GRP was conducted in the same samples used for the *In Situ Hybridization* analysis described above at 8 weeks after SCI. The distribution of immunofluorescence labeling of galanin and GRP in LSt cells was consistent with our previous reports (26, 39) and mirrored that shown for mRNA expression in both L3 and L4 spinal levels (Figure 6). Representative images in Figure 6 demonstrate the loss of GRP immunoreactivity (-ir) after SCI in both L3 and L4 spinal levels. In addition, the images show that galanin-ir remains visible in LSt cells. Quantification of the total numbers of galanin-ir cells (independent of colocalization with GRP) showed no effect of SCI in L3 or L4 spinal levels (Figure 7). However, total numbers of GRP-ir cells were reduced by SCI, an effect that was most prominent at the L4 spinal levels (*p* = 0.02; Figure 7). A statistical trend was detected (*p* = 0.054) when analysis was based on cells in L3 and L4 spinal levels combined, while no reduction was detected in L3. Due to this reduction in GRP, SCI significantly reduced the numbers of cells dual labeled for galanin and GRP, again most notably in L4 (*p* = 0.02; Figure 7). In parallel, SCI caused a significant increase in cells that were immunolabeled only for galanin (*p* = 0.026 for analysis in L3 and L4 combined, and *p* = 0.01 in L4; Figure 7). Hence, these results confirm the previously published immunofluorescence analysis and demonstrate loss GRP-ir in LSt cells, resulting in a reduction of the numbers of dual labeled GRP and galanin-ir LSt cells, and an increase in numbers of single-labeled galanin-ir LSt cells.

## Discussion

The current study provides further evidence that chronic contusion SCI disrupts expression of the neuropeptides galanin and GRP within the spinal ejaculation generator, by demonstrating that SCI reduced *galanin* and *GRP* mRNA in LSt cells. Since both galanin and GRP play key roles in the control of ejaculatory reflexes (26, 28), such reduction in mRNA expression may lead to reduced neuropeptide synthesis and release and thereby contribute to ejaculatory dysfunction following SCI.

Lumbar spinothalamic cells have thus far been demonstrated to co-express four neuropeptides, including GRP and galanin, CCK, and enkephalin. Previous studies have demonstrated that intrathecal infusions of antagonists for galanin, GRP, CCK, mu opioid, or delta opioid receptors to the lumbosacral spinal cord completely disrupt the ability to trigger ejaculatory reflexes by sensory stimulation (26–28). Conversely, intrathecal infusion of GRP or mu-opioid receptor ligands were able to trigger ejaculatory reflexes without sensory stimulation, showing that activation of these receptors is sufficient for ejaculatory reflexes (26, 27). Finally, intrathecal infusions of GRP, galanin, CCK, and mu- or delta opioid receptor ligands enhanced ejaculation upon subthreshold sensory stimulation, indicating that all receptors for the neuropeptides expressed in LSt cells are involved in the control of ejaculatory reflexes (26–28).

In a previous study, we reported that one of the neuropeptides expressed in LSt cells, GRP, was affected by SCI, while another, galanin, was seemingly intact (39). Specifically, we showed that the numbers of galanin-ir LSt cells were unaltered by SCI. However, significantly fewer galanin-ir LSt cells co-localized GRP-ir compared to sham controls, resulting in fewer dual labeled and more galanin-only labeled LSt cells. We offered two possible explanations for an effect of SCI on GRP, including epigenetic modification specifically of the *GRP* gene without affecting the *galanin* gene. But the alternative possibility which we favored was a technical one, namely that the limited sensitivity of immunofluorescence as a quantitative assay for peptide detection prevented detection of a reduction of galanin. In particular, our previous analyses based on immunofluorescence only included numbers of immunoreactive cells, whether or not immunofluorescence signal was weak or strong (39). Many factors can influence the strength of the immunofluorescent signal using antibody detection, including thickness of sections, penetration of antibodies, and location of the cell within the section. Therefore, the strength of the signal is such essays is not considered a reliable measure of neuropeptide levels (43). We also speculated that GRP peptide levels may be lower and thus more readily fall below threshold of immunofluorescent detection following SCI. Indeed, the current set of studies provides evidence for both of these claims. First, we replicated the previous findings that numbers of dual labeled cells were significantly reduced, due to a reduction of GRP-ir, while numbers of galanin-ir were not significantly impacted. These effects were most notable at the L4 spinal level. The replication of the immunofluorescence analysis was essential also because the current set of studies examined tissues at 8 weeks after SCI, while our previous studies included tissues at 5–6 weeks after injury (39) thus showing that loss of neuropeptide expression in LSt cells is not a transient event. Moreover, the current data demonstrate higher mRNA expression levels for *galani*n compared to *GRP* in LSt cells, based on a significantly higher fluorescent signal for *galanin* mRNA (4.8–4.9-fold higher than *GRP*). Finally, the current findings didn't support the hypothesis that *GRP* and galanin transcript were independently affected by SCI. It was demonstrated that SCI caused significant reductions in both *GRP* and *galanin* mRNA in LSt cells. Correlation analyses of *galanin* and *GRP* transcripts within LSt cells showed a positive relationship between these two neuropeptides under both sham and SCI conditions, suggesting that expressions of *galanin* and *GRP* are co-regulated. Thus, in conclusion, RNAscope fluorescent in situ hybridization allowed for a more sensitive analysis and demonstrated that SCI significantly impacted expression of both *galanin* and *GRP* mRNA.

Lumbar spinothalamic cells and their axons co-localize galanin and GRP, but these two neuropeptides appear to have different functional roles for the control of ejaculatory reflexes. Whereas, GRP is a potent facilitator of ejaculatory reflexes and intrathecal infusions of GRP can trigger ejaculation in the absence of sensory stimulation (26) facilitative effects of galanin infusions are dependent on concurrent stimulation of sensory inputs in the dorsal penile nerve (28). We have previously hypothesized that actions of galanin therefore may require co-release and actions of other LSt neuropeptides, such as GRP. Hence, effects of SCI on these neuropeptides may not only impact synthesis and release of the individual peptides, but also potential interactions with other LSt neuropeptides and their receptors.

Lumbar spinothalamic cells form an extended continuum or column that spans two spinal levels (21, 22, 24). Moreover, LSt cells have several know efferent targets, including cells in the intermediolateral cell column, central autonomic gray, sacral parasympathetic nucleus, sacral nucleus of the bulbocavernosus, and the parvocellular subparafascicular nucleus of the thalamus (SPFp) (17, 21, 39). However, it is unknown if there is a topographic organization within the LSt cell column for these connections. Retrograde tract tracing studies from the SPFp demonstrated labeling of LSt cells throughout both L3 and L4 spinal levels, arguing against a topographic organization (21). However, the current study showed that the impact of SCI on *galanin* transcript levels in LSt cells was more prominent in L3 cells, and failed to reach statistical significance in L4 spinal levels. In contrast, impact of SCI on *GRP* mRNA was more readily detected in L4 cells compared to L3 cells for both mRNA and immunofluorescence analyses, consistent with the finding that *GRP* transcript was lower in L4 compared to L3 levels. A possible functional relevance of topographical differences between L3 and L4 cells is currently unknown and further examination utilizing cell-specific manipulation, monosynaptic and viral tracing, and gene profiling approaches to examine specific functions and connections of LSt cells at the different spinal levels will be needed (44–47).

In studies using immunohistochemistry or fluorescence detection of galanin and GRP, dense clusters of galanin and GRP-immunoreactive axons are visualized in laminae I and II of the dorsal horn, masking reliable detection of galanin or GRP-expressing cell bodies (17, 20, 24, 26). Expression of GRP in dorsal horn is somewhat controversial with variability in ability to visualize GRP in soma likely due to differences in detection techniques using antibodies or transgenic techniques (48–50). The current study using highly sensitive RNAscope fluorescent in situ hybridization only detected low-expressing cells in dorsal horn of upper lumbar spinal levels. In the present studies, some *galanin*-expressing cells were noted in the dorsal horn of L3–4 and SCI reduced expression of galanin in these cells. To our knowledge, there have been no systemic studies examining galanin-expressing cells in the dorsal horn, and focus has been on the important role in pain of galanin expressed in dorsal root ganglion cells acting on galanin receptors in the dorsal horn (51).

In summary, the current study demonstrated that contusion injury at mid-thoracic spinal levels caused long-term reduction of *galanin* and *GRP* mRNA in the critical neuron population that comprises the spinal ejaculation generator. Since these neuropeptides are essential for control of ejaculatory reflexes, such loss may contribute to the ejaculatory dysfunction resulting SCI, and restoration of galanin or GRP synthesis and action may thus be putative treatment options. A critical future question to be addressed is to identify the mechanisms by which SCI causes a reduction of neuropeptide expression in LSt cells. It appears unlikely that these alterations are the result of acute inflammatory processes occurring at the site of injury (52–55), but rather are due to long-term changes in supraspinal influences and epigenetic modifications resulting in reduced mRNA expression of the neuropeptides (56–60).

## Data Availability Statement

The raw data supporting the conclusions of this article will be made available by the authors, without undue reservation.

## Ethics Statement

The animal study was reviewed and approved by Institutional Animal Care and Use Committee at the University of Mississippi Medical Center.

## Author Contributions

JWW: methodology, validation, formal analysis, visualization, writing-original draft, review, and editing. JS: methodology and validation. LC: conceptualization, data curation, formal analysis, methodology, validation, project administration, supervision, visualization, writing-original draft, review, and editing. All authors contributed to the article and approved the submitted version.

## Conflict of Interest

The authors declare that the research was conducted in the absence of any commercial or financial relationships that could be construed as a potential conflict of interest.
